# The Relationship between Healthcare Providers and Preventive Practices: Narratives on Access to Cancer Screening

**DOI:** 10.3390/ijerph191710942

**Published:** 2022-09-01

**Authors:** Daniela Lemmo, Maria Luisa Martino, Anna Rosa Donizzetti, Maria Francesca Freda, Daniela Caso

**Affiliations:** Department of Humanities, Federico II University, 80133 Naples, Italy

**Keywords:** preventive processes, healthcare access dimensions, providers’ narratives, cultural context sense-making, cancer screening promotion, theory-driven analysis, psychological clinical intervention

## Abstract

Cancer screening programs are public health interventions beneficial to early diagnoses and timely treatments. Despite the investment of health policies in this area, many people in the recommended age groups do not participate. While the literature is mainly focused on obstacles and factors enabling access to health services, a gap from the point of view of the target population concerns healthcare providers. Within the “Miriade” research–action project, this study aims to explore the dimensions that mediate the relationship between healthcare providers and preventive practices through the narrations of 52 referents and healthcare providers involved in breast, cervical and colorectal cancer screening. We conducted ad hoc narrative interviews and used theory-driven analysis based on Penchansky and Thomas’ conceptualization and Saurman’s integration of six dimensions of healthcare access: affordability, availability, accessibility, accommodation, acceptability and awareness. The results show that 21 thematic categories were representative of the access dimensions, and 5 thematic categories were not; thus, we have classified the latter as the dimension of affection. The results suggest trajectories through which psychological clinical intervention might be constructed concerning health, shared health decisions and access to cancer screening.

## 1. Introduction

Cancer screening programs are major public health interventions that allow the detection of precancerous conditions or early stages of disease in asymptomatic subjects in order to offer timely diagnosis and early treatments that can lead to better results.

The latest estimates indicate that about 30% of new cancer diagnoses are made during screening activities and that the long-term survival of 5 years after diagnosis is 90% in the case of breast cancer, 79% in the case of cancer of the cervix and, respectively, 65% and 66% for men and women in the case of colorectal cancer [1,2].

In Italy, cancer screenings are defined as Essential Levels of Assistance (LEA), and aim to guarantee citizens’ right to protect their health. To attain this goal, health systems are increasingly striving to ensure equity in the access to prevention services by inviting target populations to organized screening programs for breast, cervical and colorectal cancer risks. Specifically, for the risk of breast cancer, women aged 50 to 69 are invited every two years for a bilateral mammogram; for the risk of cervical cancer, women aged between 25 and 64 are invited every three years to carry out pap-smears; for the risk of colorectal cancer, people from 50 to 74 years of age are invited every two years, without distinction of sex, to undergo a test to check the presence of occult blood in their stools. However, in Italy, despite such invitations, only 41% of the population for mammography screening participate in these programs, 28% for cervical screening and 30% for colorectal screening, and these figures show a further decline if we consider southern Italy [3].

Screening should not be understood only as the provision of a test, but as a complex multifactorial process and pathway [4] consisting of the following phases: invitation, organization, possible diagnosis and treatment. The beginning of this process is characterized by the identification of the eligible subjects, the so-called target population, and continues, in the absence of early diagnosis, in a cyclical manner, inviting people to a further check in and agreed time frame.

The most recent healthcare objectives highlight the importance of consolidating these attentions on the holistic treatment of the person, taking into account that this is expressed through improved health literacy and encouraging patients to monitor their health, interacting with the health system through relationships based on trust, as mentioned in the National Plan for Prevention (PNP 2020–2025 Ministry of Health, Italy).

Within this process, the role of medical staff members is important to ensuring the effective participation in the three types of screening [5,6,7], often characterized by a general distrust in health services from the target population [8,9,10,11].

In a spirit of shared and informed decision making [12], several studies have highlighted the importance for people to have regular interaction with healthcare professionals and to receive clear and consistent information and recommendations on cancer screening [13,14,15,16,17,18,19,20,21,22,23,24], with the possibility to have a certain time and space to be heard [25] and to reveal their concerns about preventive practices [26,27,28,29,30]. From the point of view of the health professionals, it is also important to be part of a shared decision-making process. In particular, they claim the need to include both the benefits and harms of screening in the discussion and to set aside time for clarifying procedures and any false positive results [31,32,33,34,35,36,37]; they highlight poor health literacy and language barriers as the main obstacles.

In order to offer adequate prevention services, it is necessary to consider an interactive role between several levels of health professionals and patients [38].

### Access to Health Services as a Relationship between Healthcare Providers and Patient Involvement in Screening

Facilitators and obstacles to health services in the preventive setting have mostly been analyzed in the literature from the point of view of the target population concerning cancer screening [39,40]. Few studies have explored the viewpoint of providers or those who play multifaceted roles in promoting cancer screening such as advocates, educators, medical experts, quality controllers, and patients’ supporters [41].

In this work, we refer to the meaning and dimensions of access to healthcare services proposed in the theoretical framework by Penchansky and Thomas [42]. These authors examined healthcare services in terms of an adaptive relationship between providers and patients. In particular, the authors define access as the degree of adaptation between the health needs of an individual and the characteristics of the providers and the health service system: the better this adaptation, the better the access can be.

This is a key construct of health policies and research on health services which the authors define in a taxonomic way as an umbrella concept originating from a set of specific dimensions that characterize how patients adapt to the healthcare system and vice versa. In the preventive field, this framework provides a basis for examining obstacles to preventive care in unequal conditions [43] ng and looking at the aspects of the health relationship to plan effective interventions [7].

In their conceptualization of “access”, five related dimensions have been proposed: affordability, availability, accessibility, accommodation and acceptability [42].

**Affordability**: This dimension is determined by how healthcare expenses (e.g., service fees, shared health insurance costs, required payment times) are related to the patient’s perceived ability to pay for services.

**Availability**: This dimension considers the extent to which the healthcare system has the resources (for example, the adequacy of professionals, services and programs) to meet the patient’s needs.

**Accessibility**: This dimension refers to the ease with which the patient can physically access the healthcare system, taking into account factors such as transportation, distance, travel time and travel cost.

**Accommodation**: This dimension reflects the extent to which the healthcare system meets the patient’s needs and preferences, which may include opening hours and the ability to make appointments.

**Acceptability**: This dimension refers to the relationship between patients’ and health care practitioners’ attitudes, preferences and characteristics related to each other (e.g., age, sex, ethnicity, years of practice, co-morbid conditions) and to the health context (e.g., type of structure, religious affiliation).

These dimensions are independent but interconnected, and each is important in assessing access. Saurman [44] believes a sixth dimension should be integrated to the authors’ model:

**Awareness**: This dimension is an integral part of access. It is not just information and knowledge of the existence of a health service; it is about understanding and using the knowledge. It includes the realization that the service is needed, knowing who it is for, what it does, when it is available, where and how to use it, why it should be used and maintaining that knowledge over time. Awareness embraces health literacy as another component of the dimension, understood as a result of effective communication.

From a socio-constructivist perspective and a clinical approach, the aim of this work is to use interviews with healthcare providers in the Campania region to explore how they improve healthcare service access and utilization in screening programs..

Our research allows us to explore the local culture [45] in which the screening programs of the Campania region are conceived and implemented. That is, we examine the collusive emotional representations that characterize a social group towards one specific context relevant for the social group itself, in this case participation in preventive healthcare. In this respect, narration becomes a “protected and safe” device capable of promoting a look at professional experiences aimed at building meanings and points of view on daily practices. Thanks to the process of “post hoc return to experience”, narration becomes a tool to promote a process of meta-thought on the actions and daily professional practices, recovering the link between lived experiences and felt emotions. This founding process of the narrative device allows participants to put in words and rearrange experiences within the wider cultural context in which they live (function of narrative link) and within a temporal perspective that subjectively articulates the relationship between past, present and future. In this way, narration also aims to build new areas of reflection on professional experience and therefore new possible meanings on one’s subjective positioning in the relationship with professional practice [46,47,48].

Within the logic of One Health and the “Miriade” action–research project founded by the Regional Prevention Plan (PRP Campania 2020−2025 Ministry of Health, Italy) and within a qualitative research design, in this study we explore from the perspective of healthcare providers, the dimensions that mediate the relationship between cancer screening and the target population.

This study gives participants the opportunity to provide preliminary clinical reflections on implementing screening programs and on the constructing interventions to promote preventive practices that take into account the dimensions that make up the adaptation process between healthcare providers and users.

## 2. Materials and Methods

### 2.1. Recruiting of the Participants and Tools

The research was conducted as part of the broader regional research–action project (2020–2025) called Miriade: “An innovative model of research–intervention for the identification of adherence profiles to cancer screening”. This project was born within the line of evidence-based health action aimed at improving adherence to cancer screening, with the aim of building theoretical models and personalized intervention approaches to promote screening of the target population. The sample is homogeneous through intentional sampling that is specific and for which the research question is significant. The inclusion criteria for participation provided that participants worked in one of the three types of screening promoted in the Campania region (breast, cervical or colon cancer screening) and related to the public health service. In particular, we involved both oncological screening representatives and healthcare providers employed in the screening activities in each Local Health Organization (ASL) of the territory. The choice to include both referents and healthcare providers concerns the consideration that access is made up of both institutional and organizational aspects, as well as aspects related to health practices and reports. The participants were contacted on the telephone by psychologists who, after introducing the objectives and methods of the research project, interviewed them with questions about their work experience in cancer screening. All interviews were conducted between January and April 2022; they were audio-recorded and subsequently transcribed verbatim. Participant contribution was voluntary; each participant signed an informed consent for their enrolment in the study and a document for the protection of their privacy in accordance with the GDPR EU 2016/679, D.L. 101/2018. The study was conducted in accordance with the Declaration of Helsinki, and approved by the Ethics Committee of Psychological Research of the Department of Humanities of the University of Naples Federico II (Prot. N.16/22). To understand the meaning attributed by the referents and healthcare providers involved in screening services, we prepared an ad hoc narrative interview with areas to be explored and encouraging a gradual immersion in the story of one’s professional experience:

- *Professional role*: aimed at exploring professional history and specific belonging to one’s work context. (How and for how long have you been involved in cancer screening?)
- *Description of the territory*: aimed at exploring the relationship of the territory in its structural, spatial and geographical aspects. (Based on your experience, can you describe the reference area of this ASL?)
- *Representation and relationship with the target population*: aimed at exploring emotional facets of the relationship with the target population and specific characteristics of the health relationship (Could you describe with three adjectives, words or images the audience you deal with?)
- *In deep episodic narratives*: aimed at exploring fragments, memories and experiences significant for oneself and for one’s professional role that have characterized the healthcare relationship in preventive practices. (In the course of your experience, do you remember a particularly significant event in the performance of your professional practice?)
- *In deep low episodic narratives*: aimed at exploring fragments, memories and experiences that are meaningful to oneself and particularly difficult/critical in one’s professional experience (Can you tell how you managed if there was a time when you had to communicate a negative outcome? Have you ever had to use compelling arguments to promote screening?)
- *Regulatory health practices of the screening decision-making process*: aimed at exploring the point of view of oncological screening representatives in planning management policies and screening practices, constraints, resources and transformative ideas underlying the promotion of adherence to screening practices. (What strategies have already been adopted by the health services of this area to implement screening? What other health and social practices, in your opinion, could facilitate the participation of women and men in your area in terms of prevention?)

The entire interview took an average time of 20 min.

### 2.2. Data Analysis

The coding of the narrative corpus was conducted through a theory-driven approach.

The analysis was conducted by 3 independent judges and proceeded through two phases. First, the texts were analyzed using predefined categories derived from Penchansky and Thomas’ conceptualization [42] and Saurman’s integration [44] about 6 dimensions of healthcare access: accessibility, affordability, availability, accommodation, acceptability, and awareness (a top-down approach); this allowed further unexpected themes to emerge (a bottom-up approach). The steps of analysis are:

(1) Using 6 theoretical categories line-by-line coding of all narrative transcripts from which the main groups of themes with common meanings were identified.
(2) The classified themes were inserted into the conceptual structure based on the relationship and connection of the theme to the access components.
(3) The emergence of unexpected themes from the narratives, in particular, specific ways in which the narratives articulated emotional and relational levels of healthcare access did not derive from the predefined theoretical categories, but they have been grouped into a new category.
(4) Each dimension of access was discussed in relation to its thematic content, capturing any difference between the types of screening.

The 3 judges, (D.L.; M.L.M.; A.R.D.) experts in qualitative data analysis, independently examined the entire textual corpus building their coding system. Disagreement between judges was managed through ad hoc meetings in order to identify superordinate categories capable of reducing the range of disagreement. The reliability can be calculated by dividing the number of agreements by the total number of agreements plus disagreements. The final coding of all interviews reached 90% agreement on the coding of all interviews.

## 3. Results

### 3.1. Subjects’ Characteristics

Among 52 oncological screening representatives and healthcare providers in cancer screening practices enrolled in this study (24 males; 28 female; Mage = 53); 15 were referents (29%); 13 were radiology technicians involved in mammography exams (25%); 17 were obstetricians and/or gynecologists involved in pap tests (33%); 7 were laboratory experts, pathologists or colonoscopy operators involved in the prevention of colorectal cancer (13%).

### 3.2. Access Dimensions and Categories

The narrations highlight a total of 21 thematic categories which are representative of the six theoretical dimensions of access. However, five thematic categories, on the other hand, do not meet the necessary requirements to fall within the dimensions proposed by the authors. Thus, we created a seventh theoretical dimension—affection—which appears to us to be characteristic of an emotional and relational level about the adaptation between the target population and the health system with regard to oncological preventive practices.

The thematic categories emerged from the analysis are the following (Table 1).

#### 3.2.1. Affordability

Three thematic categories fall into this dimension which concerns the economic accessibility of the health service in cancer screening practices and the users’ perception regarding this aspect.

(1) The free offer: inclusion and experiences of exclusion.

This category highlights how cancer screening practices are completely accessible economically for users and respond to the criterion of health equity; indeed, they are provided free of charge by the National Health System (NHS) in order to promote inclusive healthcare and overcoming economic barriers. This element is related to the perceived ability of users to both know and appreciate this gratuity. The narratives reveal the users’ mistrust in the public offer, due to the belief that something that is not paid for has no value and is not sufficiently professional and valid, characterized by an impersonal aspect which undermines warranty and reliability. On this point, the providers emphasise the high quality of the public context thanks to excellent professionals and the use of the latest technologies, considered as elements of great importance.

*“I do not know why there is such a strong mistrust in the public system; I have worked both in the private and public sectors and I can assure you that you work much better in the public one. You are less frustrated and, above all, the quantity of work is proportional to the staff; moreover, the equipment is state of the art. Many patients, instead, have opposite views—“We prefer the private sector because, you know, if you pay you have a better service”. On the contrary, the greatest disservice probably occurs in private facilities, where staff are frustrated, underpaid and with a lot of work to do. The public system can be a guarantee, and it is even linked to free services”* (healthcare provider, woman, breast cancer screening).

(2) Public health services: the underestimated value of the institutional network.

This category highlights how the oncological screening activity within the public service is part of a public network logic which, from their point of view, facilitates and guarantees the passage from a first to a second level of screening, possibly up to a third, not abandoning the subjects who can thus feel they are on an accompanied pathway. The important aspect is that screening remains a territorial competence, therefore managed by health districts that deal with the first level, so that hospitals remain dedicated to major levels of care.

*“The public system has a network, and it is a very important thing because any step that must be taken, even if in another region, is always public and so there is a relationship that is established directly from the structure. Instead, a private system is limited and can operate only up to a certain point: if a pap test by a private gynecologist results to be positive, the doctor must indicate where to g,o and this is a second level not linked to the first”* (referent, man, cervical cancer screening).

(3) Economic investments for a digitalization of healthcare.

This category highlights how the protection of citizens’ health is a public issue and, cancer prevention is a health objective maintained through health monitoring activities. Therefore, the NHS uses economic resources both to create dedicated spaces, such as within the clinics for cervical screening, and to invest in digitizing processes and services in the health sector through the construction of computerized records.

*“We have a platform, called Saniarp, where we record pap tests and where the analysis laboratory registers the positivity. This platform should be managed first of all by the general practitioner, whose alert system can notice the positivity of a patient”* (referent, woman, breast cancer screening).

#### 3.2.2. Availability

Four thematic categories fall into this dimension which concerns the extent to which the health system has adequate resources to meet the users’ needs in the context of screening.

(1) The formative and specific gap: towards healthcare operators devoted to screening praxis.

This category highlights the need for training for all health professionals who deal with the population and screening users, so that this may become a driving force for the promotion and maintenance of a good relationship. This training should involve the specific level of knowledge regarding the programs, as well as relational and communication plans.

The narratives reveal that the experience of a competent, reliable health system providing adequate answers creates easier adherence.

*“Anyone working on screening programs should join and promote membership. This is why training is needed, and it must involve all the healthcare staff. Furthermore, in order to guarantee the test to everyone, resources must be offered especially in terms of the number of operators and training. There is no trained staff or communication skills. We must promote membership and prevent people from leaving”* (healthcare provider, woman, colorectal cancer screening).

(2) The co-construction of a fidelity process.

This category highlights the importance of screening in terms of the process and the path the patient follows. In this field, there are numerous strategies that cross the three stages of prevention: the early one, which concerns the promotion and invitation plan; the mid one, which is the time of tests and examinations; the late one, understood both as the time for communicating the outcome and as a possible passage to a second level. The healthcare providers feel they are accompanying users when they contact them by phone when they are already known, at the time of reservations and in the subsequent response phases.

*“Calling them, contacting them directly, booking, calling them again if an appointment is required. We have to provide a service from the booking to the delivery of the organized report. [...] We get the numbers rightly from the health service; consequently, we send them to women who are of screening age and therefore both for the first time and after three years; in short, it depends, therefore also to new women of course, every time we provide new addresses because many leave, many come to live here, so let us say it is dynamic and let us say that the regional registry helps us a lot in this”* (healthcare provider, woman, cervical cancer screening).

(3) From persuasion to the construction of competent users.

This category highlights the numerous strategies aimed at offering screening services: dedicated days, service openings on days and times other than those established, trucks, facilities of all kinds, personalized invitations, hpv-test in the pharmacy. The narratives on this theme reveal the ambivalence between a super-offer given everywhere aimed at deleting all boundaries, both physical and psychic, and the need for a space in which people can build their own subjective motivation in choosing to adhere to the preventive practices but above all to maintain them over time. In the absence of a demand for prevention, the healthcare providers emphasize the need to differentiate the health offer from all other types of offers to the population. Promotion and enlistment should not be confused with persuasion because people need to be motivated to do so.

*“It must become a person’s own decision to undergo screening, as an appointment. The important thing is precisely to make this empowerment grow within the person; otherwise we can send all the paper invitations in the world, but if the motivation is not born within the subject it iss a bit as if everything we do would fail, right”?* (healthcare provider, woman, colorectal cancer screening).

(4) Monitoring of preventive practices for an engaging service.

This category highlights healthcare providers’ competence to meta-reflect on the preventive practices that regulate the health system and to compare them with those that may be more efficient, with the aim of maintaining the population’s adherence to screening practices. For example, the use of reviews and monitoring the progress of individual districts is a way to support the engagement of citizens and plan ongoing improvement actions. In addition to the strategies already highlighted to reduce access difficulties, another important example is given by the delivery of the CD of the breast ultrasound test: the public service usually releases only the result of positivity or negativity. Instead, it is important for the construction of a story to providw a more in-depth result to the user, with the disk and a clear report that marks and highlights any inflammation or fibroadenoma.

*“In the meetings with the district directors and the managers, obviously we always try to follow the data, so if a district lags a little behind, you can immediately see it from the statistics. Then you try to understand what iss wrong with the outpatient clinic in order to try to solve the problem. So we always try to balance things out thanks to the many meetings that are regularly held”* (referent, man, mammography, cervical and colorectal screening).

#### 3.2.3. Accessibility

Three thematic categories fall into this dimension, which concerns the ease with which the user can physically access the health system taking into account factors such as transport, distance, travel time and travel cost.

(1) The risk of exclusion: logistic barriers.

This category highlights the logistical barriers between rural and mountain areas and the screening centers that are mostly found in urban centers. Even in the cities, districts can often be far from residential areas that are not connected and connectable to health services. The provinces of Campania cover very large territories for which a single district is the reference for a very large population.

*“To get there by public transport is difficult here, because we do not have a bus service. There used to be a bus stop, I do not know why it was removed...How can they get here?...Without a car it is a long walk. We must also consider an important aspect: this is a very vast territory. Let us say that it encompasses four districts”* (healthcare operator, man, mammography screening).

(2) An itinerant and iterative prevention

This category highlights the mobile prevention at the user’s proximal service. In particular, the traveling reference is to prevention vans or mobile clinics that carry out itinerant activities dedicated to screening for early diagnosis. These are totally autonomous vehicles that can reach any location and be positioned in main squares to make prevention accessible to all. Specifically, they refer to the screening of the uterine cervix, as the necessary material and machines are part of the van’s equipment, and this aspect is also seen as a way to involve users in other types of screening. Another aspect highlighted by the operators is the iterative aspect of the promotion: it may seem that a “spot awareness” is not effective, but it must be a capillary work to be repeated cyclically.

*“Because by taking it to the streets, people do not have to go to the ASL (public health center); they find it nearby as it is the ASL that goes to the people. The van was a very important device, considering the number of women who underwent check-ups for the first time in the van and then came to our structure for the screening”* (referent, man, cervical cancer screening).

(3) The over-district between facilities and the risk of overload.

This category mentions, among the most effective strategies, the need to facilitate access to screening services, to remove the district constraint or that people who live in a specific neighborhood refer to the health district of that area. At the same time, this element brings about the risk of overload in some districts that may have to manage a disproportionate number of users compared to other districts.

*“People commute, live in one place and work in another, so we did not intend to set territorial limits so that women could easily take mammography tests throughout the company’s territory regardless of the district to which they belong [...] even if it would be preferable for each district to deal with its own area of competence, because, for example, the central ones risk to be overbooked; we must be spread throughout the territory and above all have an identical modus operandi”* (referent, woman, mammography, cervical and colorectal cancer screening).

#### 3.2.4. Accommodation

Three thematic categories fall into this dimension, which reflects the extent to which the healthcare system meets the patient’s needs and preferences, which may include opening hours and the ability to make appointments.

(1) The bureaucratic facilitation of booking procedures.

This category highlights healthcare providers’ ways to meet the user’s need to have a simple booking procedure and avoid waiting for a long time to arrange an appointment. In fact, the screening services can be managed by the regional and corporate Single Booking Center (CUP), accessible in the pharmacy, at the general practitioner’s office and soon on their own App. Furthermore, when possible, operators prefer to ease these processes, even by booking themselves at their workplace.

*“We included the mammography screening service in the Single Booking Center so that women can access the CUP anywhere, also in pharmacies, and we pay the EUR 2 fee for the reservation. Even the general practitioner, connected to the corporate and regional CUP, can book women directly for mammography wherever they choose to go and soon the App message will arrive on their mobile phone [...]If a woman has problems at the CUP to wait for the reservation, I go there and book it myself. We need to reduce personal bureaucracy issues, because we are always in a hurry, especially women”* (referent, man, mammography screening).

(2) A non-stop service that takes care of quality.

In the exploration of the possible causes hindering the user’s active decision-making process to participate in cancer screening, the reduction and lowering of the “borderline” between service opening and user needs represents a narrated area (together with the other strategies implemented as the abovementioned over-district, vans, etc.). A peculiar aspect becomes that of offering the possibility of screening in an “open” way, in the afternoon and on the weekend in order to facilitate the work needs of users, but taking care of the places and rooms that should always be kept clean and tidy.

*“They have to come every day, and we accept them both in the morning and afternoon, so those who are busy in the morning can come in the afternoon, while those who are busy in the afternoon can come in the morning. I have a nice pink room to welcome them”* (healthcare provider, woman, cervical cancer screening).

(3) Interventions by operators in the management of the waiting time for results.

This category highlights how the response time of the screening results represents a limit that does not satisfy the users’ need to know how they are. Therefore, the screening operator, although not in charge of communicating the results, takes the responsibility of an anticipation aimed at reassuring the user.

*“Three weeks to get a response is too long... we anticipate it, even if we are not doctors in charge of diagnosis—obviously we do not give you the report immediately because we need two radiologists to analyze the exam, but we give you feedback. We tell them to sit in the waiting room after the exam, and with the radiologist on site we check the test. If the patient is completely negative, we dismiss her and she goes home relieved; instead, if we have a diagnostic doubt, if we can we integrate her at the moment, otherwise we give her an appointment after a few days in order to limit the anguish of the phone call; indeed, our patients are terrified of receiving the in-depth phone call”* (healthcare provider, woman, breast cancer screening).

#### 3.2.5. Acceptability

Four thematic categories fall into this dimension, which reflects the relationship between the attitudes, preferences and characteristics of the patient and the healthcare provider related to each other (e.g., age, sex, ethnicity, years of practice, co-morbid conditions) and to the health context (e.g., type of facility, religious affiliation).

(1) Healthcare operator’s gender role within screening exams.

This category reflects on the sex of the operator in cancer screening practices. Male doctors notice some embarrassment in women who are going to undergo mammography and a pap-test; they try to be polite and sensitive so that the healthcare provider is seen exclusively in his/her role. This allows women to better understand the professionalism of the doctor. Operators, on the other hand, tell of the relief declared by women when they find themselves in front of a female doctor.

*“Women are very reluctant to take an exam: first of all, a woman is modest. However, she has to get undressed. In my case it is not easy, as I am a man; you have to be sensitive and make the woman understand that there and then you are a health worker. Saying it is not enough; a woman has the sensitivity to perceive when someone is making you feel at ease; instead, when you say it just to chat, it does not work”* (healthcare provider, man, breast cancer screening).

(2) Meetings between ethnic groups and health equity.

This category describes the good practices implemented for the benefit of the most vulnerable groups at the local level with the aim of tackling health inequalities. Some health districts in particular, especially those in the Naples city center, are more characterized by multi-ethnicity. Health is intended as a resource for the community, and all citizens must be guaranteed equal access to its care.

*“Our district is characterized by multi-ethnicity and is also a reference point to various non-profit organizations with which we collaborate. There was also surveillance on Nigerian victims of trafficking. Then there was an outpatient clinic for transgender people, also for those who wanted to undergo gender change and then… there were many of them from Eastern Europe and many Africans”* (healthcare provider, woman, cervical cancer screening).

(3) Global taking charge of the feminine in the consultation framework.

This category, in particular about cervical cancer screening, highlights how preventive activity is deeply connected to the care of women’s health, the founding purpose of counseling clinic centers. In other words, healthcare providers underline that the practice of oncological screening enters a framework of wider meaning in which women can feel the clinic is a place that follows them in all the stages of development and health.

*“Women obviously feel a little more taken in... a real, efficient management. Instead, taking charge of the exam alone becomes a deterrent in the end, because they may say “ Well, I am going there, I am just doing that, why should I”? On the contrary, I have more chances of check-up there, […] not only in the genital sphere but also, why not, in the psychological sphere of… of the woman in menopause, of the girl who wants to use contraception and so on. Thus, offering other incentives apart from screening, in my opinion, is just the total taking charge of… the health prevention of women”* (healthcare provider, woman, cervical cancer screening).

(4) The generation gap in adhering to screening

This category highlights the importance of the age factor as a determinant of adherence to screening. While young women seem to favor participation in prevention, there is more resistance in those aged between 50 and 60, who appear very frightened of any type of manipulation that concerns the investigation of the intimacy of their own body.

*“Perhaps the group on which we should focus is precisely that of the so-called old school, women in their fifties or sixties; they are reluctant, especially those in our neighborhood, because they are afraid, they are terrified and cannot access precisely out of terror. Young women, on the other hand, are a little more courageous!”* (healthcare provider, woman, cervical cancer screening).

#### 3.2.6. Awareness

Four thematic categories fall into this dimension, which concerns knowledge on services, the ability to use this knowledge while maintaining it over time and the citizens’ health literacy as a result of the relationship and communication with health professionals.

(1) Acknowledgement of screening programs.

This category highlights the healthcare providers’ belief that poor adherence to screening is due to lack of knowledge on the part of the target population. People do not know that the health company organizes such programs; they do not know that the visits are free, and it is only after the first contact with the facility that it is possible to understand what it is and maintain this knowledge over time. In particular, healthcare providers report little knowledge on colorectal screening, as well as great confusion and false beliefs on the papilloma virus. On this point, the role of the MMG (doctor of general medicine) emerges; they should perform, according to the operators, a primary function in promoting screening.

*“People are poorly informed... Information is not homogeneous in the area; many women do not know about the program; many women are surprised to have access to mammography every two years because there is false information in the area. Some people do it only because of word of mouth. As for the doctor of general medicine…zero, completely zero”* (healthcare provider, man, breast cancer screening).

(2) Word of mouth to women: a way to increase health literacy.

This category highlights how healthcare providers derive from their experience in the field a useful way to promote health knowledge and literacy, that is to give women who use the service the role of health promoters for other women. Witnesses of a possible positive experience in the health district, women can bring out useful and necessary information to make preventive choices.

*“Yes, because they stay for a long time, they are really happy when they leave, so we say—please tell your friends and relatives that we are here at your disposal for free mammograms, from 45 to 69 years old, without obligation, without any cost, and we say so every time just to find more people to take here”* (healthcare provider, woman, breast cancer screening).

(3) An unknown and neglected body.

This category underlines the value of screening in terms of health monitoring and control of any pre-cancerous conditions in asymptomatic individuals. In the context of screenings, the body does not present any symptoms, but in particular in the context of cervical screening, gynecologists are struck by women’s lack of knowledge about their body and its functioning, as well as the habit mixed with resignation that there may be vaginal changes or inflammations that are not supposed to be taken care of.

*“What we notice is that certain situations at the vaginal level should not be such, because they are in any case a sign of an alteration, even simply a vaginosis. Therefore, an alteration of what is the normal bacterial flora, even the most consistent losses are sometimes almost accepted as normal. The continuity of these things is not good for you... women tell you “However, it is always like this”, as if it were a fact; instead, then they realize that taking just a little care, the situation changes. So, people have certain troubles that are taken for granted.... Simply the pap test is sometimes an opportunity to find out changes that are not normal; however, when midwives perform the pap test and find some anomalies, they actually agitate for the visit. Therefore, the attractiveness can be the pap test, but then the result can be expanded”* (referent, man, cervical cancer screening).

(4) Between technique and transmissibility: health communication.

This category shows that it is clear that health literacy is promoted more within the health relationship. Therefore, healthcare communications, both about the promotion and about the results of the screening exam, must be carried out in a technical but communicable manner. In each territory, healthcare providers find the most suitable *language to pass clear and effective news to the population.*

*“The explanation must be technical, yet it must be understood. The problem is to reassure women that having a non-negative pap test means that something must be done so that in 5–10 years conditions will not be worse. The idea is to give a woman peace of mind in relation to the positivity of an exam that regards tumors to make it clear that the response tells us that there is a lesion which is a much earlier step than the tumor”* (healthcare provider, man, cervical cancer screening).

#### 3.2.7. Affection

Five thematic categories fall into what we have defined as affection, defined ad hoc since they are not attributable to the theoretical dimensions of access aforementioned. We could describe this dimension as the emotional and relational quality of the health relationship whose adaptation regulates the access to screening services. In other words, in the narratives of healthcare providers involved in screenings, it is configured as the affective dimension that crosses the relationship between the representations of their preventive practices in the oncology field and the representations of the user’s experiences. The thematic categories of this dimension are:

(1) Relational strategies during the screening exam.

This category brings into play the affective matrix of the healthcare providers’ way of approaching users during screening exams. Through what they describe as “distraction”, they bring the person, who is a patient at that moment, back to her subjective condition, to her habits, to the things she likes to do most, reminding them they are also a person during the exam, not only someone at risk of illness.

*“You have to treat her patiently during the exam, and distract her. Maybe the patient is asked “How is it going? Do you have children? What do your children do with you”? We ask her if she knows how to cook and how she makes pasta and potatoes. You have to distract her during the exam. We have a chat as if we were having coffee together., She is distracted and does not experience strong tensions”* (healthcare provider, man, breast cancer screening).

(2) Specific emotional representations of the three screening exams.

This category offers a representation of specific affective qualities in relation to the three types of screening tests that affect one’s sexual characteristics. With mammography, women have to undress and allow the X-ray technician to position their breasts for the test. Showing one’s private parts in the gynecological position creates reticence. Regarding colorectal screening, the first level examination, i.e., the collection of feces in a search for occult blood, is considered by operators as an autonomous and simple action because it is linked to defecation, a natural act that takes place every day in one’s own home. What has a significantly affective matrix in this type of screening is the terror of colonoscopy, a test feared for its invasiveness.

*“To undergo mammography, women have to take off even their underwear. They have to show their breasts for the mammogram... As for the pap test, it is the position that creates the woman’s reticence, not the examination itself. Regarding the colon test, on the other hand, I think that people’s fear is of the second level, that is colonoscopy. We perform sedation, but we cannot have an anesthetist always there, so it is conscious sedation”* (referent, man, mammography, cervical and colorectal cancer screening).

(3) Anxieties and fears: emotional space within a healthcare relationship.

This category brings into play the emotional and relational difficulties of both healthcare providers and consumers of the cancer screening services. Significant situations and specific memories of events are brought up in the narratives to think about the healthcare providers’ skills both during exams and in communicating results. Another relevant aspect in emotional terms is dealing with users’ previous negative experiences, trying to make a difference and provide a different type of experience.

*“There was a patient who had such severe anxiety, not for the exam but for fear of discovering something wrong, that she vomited during the exam. She was so nervous and tense that it was really difficult to be able to calm her down. She really caused me trouble, honestly. I mean, seeing patients who are so agitated and pale that they start vomiting after the first compression, and some even faint during the exam, these are somewhat particular situations; it is difficult to know how to behave, also because nobody teaches us. Perhaps this would be very interesting to investigate, that is, how to treat patients’ anxiety”* (healthcare provider, woman, breast cancer screening).

(4) Prevention as self-love.

This category highlights the emotional meaning of prevention, understood as attention and love for oneself. Failure to adhere to preventive practices is in fact considered as lack of love for oneself and as a defense against the risk of illness.

*“The problem is that these women often tend not to love themselves, in my opinion; they receive the letter but disregard it. In the end, we must love ourselves., We have a life, and we must respect this life that has been given to us. Consequently, we must take care of ourselves, and the only way is prevention. There is a big difference between tackling a tumor or a neoplasm at the beginning and when, unfortunately, it has already spread everywhere. This is wrong”* (referent, woman, breast cancer screening).

(5) Fear of discovery illnesses and block of action.

This category recognizes that the fears underlying the non-participation in cancer screening relate to illness and death. Avoidance is connected to the fear of discovering that you have, although in an initial stage, an illness such as cancer that opens up the deepest fears related to death.

*“In my opinion, the emotional obstacle is not to be under-evaluated. Indeed, I think it is one of the most important; beyond the fact of not wanting to come, not to be seen and things like these, emotion plays an important role. There is a lot of fear, so they prefer not to know—I am afraid, I do not want to know”* (healthcare provider, woman, colorectal cancer screening).

## 4. Discussion

The thematic categories that emerged from the analysis of interviews with referents and healthcare providers involved in cancer screening are attributable to the six theoretical dimensions that describe access to health services, as mentioned in the literature. It was also necessary to identify a seventh dimension which, in our opinion, characterizes the adaptation relationship between the cancer screening target population and the health system in cancer screening; we have defined it as “affection”, thus putting in place the affective matrix narrated by the healthcare providers regarding their preventive practices and users’ responses.

From a psychological and clinical point of view, using this model allows us to reflect on the clinical and intervention barriers and implications of our study, which was designed to improve participation in preventive practices. The clinical reflection arises from an integrated reading of the different dimensions explored starting from the analysis of the narratives. The analyzed narratives make it possible to highlight that to ensure affordable screening, it will be necessary to enhance the public perception of the service. An element that differs from other studies with respect to the affordability dimension is that the screening programs in the national health system are free and do not require health insurance [43], an element that is configured as a barrier in the literature [49].

For a service to be available, the theoretical model indicates that it must meet the needs of users and of the communities served [44]. We think that the preventive services being offered by the national health service meet this parameter. What our results show is that there is an absence of demand for screenings despite numerous offers at every stage aimed at promoting loyalty, engagement and subjectivation with respect to the preventive process and health. These offers are declined, in particular, for colorectal screening because, according to the operators it is the least known, screening. As recommended by the literature [50], training for service operators is a necessary resource to use in the quest to build relationships and involvement in healthcare services.

An accessible service brings into play the logistical aspects and therefore the need to be close to the consumer in terms of time and distance. In line with other international studies, there are also strategies in the field of cancer prevention in the Campania region to bring prevention to people’s homes through itinerant vans or through the free opening of districts without the constraint of being a resident of the territory. In particular, for cervical and colorectal cancer screening, itinerant prevention is possible because the tests do not include machines that are too large, as in the case of mammography.

An adequate service is well organized to accommodate users who can become competent in the use of the service itself. For this to happen, providers and patients commit to navigating bureaucratic practices that often discourage patient participation. Providers can also offer a welcome that exceeds time limits, taking care that the facilities provide welcoming and safe environments suitable for women and men at every stage of their life [51,52,53,54].

The results also show that in order to ensure an acceptable service, we must take into account attitudes and characteristics of both the operator and the user, as well as the relationship between them. While in the study by Aleshire et al. [43] this dimension mostly takes into consideration aspects of the user’s beliefs, our results highlight the influence of the operator’s sex, the user’s age range and cultural differences in the relationship that promotes adherence to screening. As in other studies, it is important that suppliers develop a relationship of trust and collaboratively involve users in the healthcare decision-making process [52,55,56] Healthcare professionals should be respectful, non-judgmental and welcoming to make users feel at ease and listened to [52,56,57,58]. This access dimension includes female screening—breast and cervical cancer screening—regarding feminine characteristics to be considered in order to render an acceptable service.

A service must ensure that information is readily available to all users and that this information is always kept up-to-date. Moreover, the service needs to articulate its effective communication and information strategies that, in this case, include health literacy. The main problem in the field of screening is that people are not aware of the programs and, even worse, do not know their own body, its functioning and risks. The female body emerges as unknown to users and often poorly cared for and unprotected. We found it useful to use the dimension proposed by Sauman [44], and we highlight that; we also highlight that dealing with awareness does not refer only to the level of information and knowledge, but they need to be transmitted in simple and effective ways so they can be maintained over time. As highlighted in the literature, if they are aware of the local context and of the needs of the population, service operators can provide more appropriate and effective care, and patients can access and use these services better if only they were aware of it in the first place. The awareness figure also emerges in other studies as a missing item with respect to the user’s awareness of the structure, service and its functions [59].

Self-care and attention to the signals of one’s own body cannot be intermittent but must intertwine the information and knowledge level with the emotional one [60].

The addition to the model of the dimension of affection represents the integration of the affective matrix that acts as a regulator of the subject’s thought and behavior in relation to preventive practice choices and access to health services. Decision making, as well as the plan of individual motivation to act in the context of preventive processes, is a question that arouses specific reactions, both in health professionals and in patients, in the presence of fears and anxieties related to examinations, risk of disease and death. An affective service thus allows us to consolidate attention holistically on the person.

We believe that the seven dimensions explored can guarantee the construction of clinical psychological intervention strategies only if they are interpreted and read in an integrated way.. The contextual, cultural and organizational aspects of healthcare acess (affordability, availability, accessibility and accommodation) intersect with the aspects most linked to an intrapsychic and socio-relational plan (awareness, affection and acceptability). Together they can build intervention strategies useful for the implementation of screening program access.

## 5. Conclusions

According to our study, access to preventive cancer screening services in the Campania region is regulated by seven dimensions. These dimensions involve system issues, cultural issues and health relationship issues, influencing the use of care and the shared decision-making process in preventive practices. This expanded model allows us to take into account the limits and resources of the territorial services, the strategies implemented for the promotion of screening and the relational and emotional specificities that allow shared health decisions.

Despite the limitations of the study, mainly due to the limited sample representative of a single Italian region, makes it difficult to generalize the results, these reflections suggest some trajectories from the point of view of clinical intervention which, in our opinion, present interesting challenges and opportunities.

Clinical psychology traditionally emphasises the question that subjects pose to themselves when confronting a need or a discomfort; in this context, instead, clinical psychology faces the challenge connected to constructing a subjectivized and autonomous question of subjects in the absence of discomfort and aimed at monitoring their own health.

In this sense, aspects of psychological-clinical epistemology come into play, such as: the relationship with the strands of subjective human temporality—past, present and future; the processes of signification, as mediators, of the relationship between the subject and the relationship with concepts such as health, risk and disease; the relationship and perception embodied with identity and body awareness; the role of defense or promotion of action played by negative emotions such as terror, fear, anxiety, distrust and disgust [60].

Within the socio-constructivist framework of this work, these aspects can be transformative only if they are conceived as intertwining with the contextual and therefore relational culture in which they evolve and moving in a continuous process of definitions and re-definitions generated from the culture in which they are produced. In this sense, the intertwining cannot ignore the organizational, structural and social aspects involved in the investigated area.

Therefore, the effectiveness of cancer screening programs should not only be connected to the increase in the number of screening tests, but as the result of a synergistic exchange between referents, operators, local culture, territorial context and subjects, setting for itself a broader objective of the construction of subjects capable of acting for their own health and that of the community.

## Figures and Tables

**Table 1:** Healthcare access dimensions and access categories in cancer screening programs.

Healthcare Access Dimensions	Access Categories in Cancer Screening Programs
*** AFFORDABILITY ***	*The free offer: between inclusion and experiences of exclusion* *Public health services: the underestimated value of the institutional network* *Economic investments for a digitalization of health.*
***AVAILABILITY***	*The formative and specific gap: towards healthcare operators devoted to screening praxis* *The co-construction of a fidelity process* *From persuasion to the construction of competent users* *Monitoring of preventive practices for an engaging service*
***ACCESSIBILITY***	*The risk of exclusion: logistic barriers* *An itinerant and iterative prevention* *The over-district between facilities and the risk of flooding*
***ACCOMMODATION***	*The bureaucratic facilitation of booking procedures* *A non-stop service that takes care of quality* *Interventions by operators in the management of waiting for results*
***ACCEPTABILITY***	*Healthcare operator gender role within screening exams* *Meetings between ethnic groups and health equity* *Global taking charge of the feminine in the consultation framework* *The generation gap in adhering to screening*
***AWARENESS***	*Acknowledgement of screening programs* *Word of mouth to women: a way to increase health literacy* *An unknown and neglected body* *Between technique and transmissibility: health communication*
***AFFECTION***	*Relational strategies during the screening exam* *Specific emotional representations of the three screening exams* *Anxieties and fears: emotional space within healthcare relationship* *Prevention as self-love* *Fear of illness discovery and blocking of action*

## Data Availability

The data are available writing to the corresponding author.
